# Reviewers Index

**DOI:** 10.3400/avd.ri.23-01000

**Published:** 2023-12-25

(The reviewers who completed the peer-review for manuscripts submitted during Nov 1, 2022–Oct 31, 2023.)

The seven reviewers written in boldface reviewed more than 4 papers.

Annals of Vascular Diseases do thank all the reviewers for their strong support and cooperation.

## A

Akagi, Daisuke

**Akamatsu, Daijirou**

**Akasaka, Junetsu**

Akasaka, Kazumi

Al-Salman, Mussaad

Altarazi, Louay

**Anai, Hiroshi**

**Anzai, Hitoshi**

## B

Banno, Hiroshi

Bashar, Abul Hasan

## C

Cheng, Stephen

Christoforou, Panagitsa

## D

Daitoku, Kazuyuki

## F

Fujii, Takeshiro

Fukuda, Ikuo

Fukuda, Hirotsugu

Furuyama, Tadashi

## G

Goto, Hitoshi

Guntani, Atsushi

## H

Hamamoto, Masaki

Harada, Hirohisa

Hashimoto, Takuya

Hata, Taishi

Hattori, Tsutomu

Hayashi, Shin-Ichiro

Hiraoka, Arudo

Hirata, Mitsuhiro

Hiromatsu, Shinichi

Hirose, Hitoshi

Hosaka, Akihiro

## I

Ihara, Tsutomu

**Iida, Yasunori**

Imai, Takahiro

Inaba, Masashi

Inaba, Yu

Inuzuka, Kazunori

Ishibashi, Hiroyuki

Ito, Hiroyuki

Iwakoshi, Shinichi

Iwata, Hirohide

Izumi, Yuichi

Izutani, Hironori

## J

Jibiki, Masatoshi

## K

Kadohama, Takayuki

Kamal, Dhafer

Kanaoka, Yuji

Kaneko, Kenjiro

Kawaharada, Nobuyoshi

Kawai, Yoshiko

Khan, Mohammad

Kikuchi, Shinsuke

**Kitagawa, Atsushi**

Kodama, Akio

Kodera, Aiko

Komai, Hiroyoshi

Kondo, Norihiro

Kondo, Yuka

Kritayakirana, Kritaya

Kudo, Toshifumi

Kuma, Sosei

Kunihara, Takashi

Kurimoto, Yoshihiko

## L

Lee, Byung-Boong

## M

Maemura, Koji

Matsubara, Kentaro

Matsubara, Shinobu

Matsuda, Hitoshi

Matsui, Yoshiro

Matsumoto, Harunobu

Matsumoto, Takuya

Matsushita, Masahiro

Matsuyama, Katsuhiko

Minatoya, Kenji

Mine, Takahiko

Mitsuoka, Akito

Mitsuoka, Hiroshi

Miyata, Tetsuro

Mo, Makoto

Morikage, Noriyasu

Morishita, Eriko

Morota, Tetsuro

Mousa, Ahmed

## N

Nakamura, Takashi

Nishibe, Toshiya

Nomura, Tadashi

## O

**Obara, Hideaki**

Okada, Kenji

Okazaki, Jin

Orihashi, Kazumasa

Otani, Norifumi

Otsui, Kazunori

## P

Paocharoen, Veeraya

Piffaretti, Gabriele

Pinjala, Ramakrishna

## R

Rehman, Zia

## S

Sakuda, Hitoshi

Sano, Masaki

Sano, Motoaki

Sata, Masataka

Sato, Osamu

Seike, Yoshimasa

Shibata, Tsuyoshi

Shigematsu, Kunihiro

Shimamura, Kazuo

Shimizu, Tsuyoshi

Shindo, Shunya

Shirasu, Takuro

Sotoda, Yoko

Sugano, Norihide

Sugimoto, Takaki

Sumi, Makoto

Suzuki, Shinichi

## T

Takano, Hiroshi

Takayama, Toshio

Takayama, Yutaka

Takayama, Katsutoshi

Taniguchi, Ryosuke

Ting, Albert

Tomaru, Takanobu

Toya, Naoki

Tsuji, Akihiro

Tsukube, Takuro

## U

Uchida, Tetsuro

Uchida, Keiji

Ueda, Tatsuo

Ueyama, Keishi

Unno, Naoki

## V

Vardhan, Vivek

## W

Wada, Hideichi

Washiyama, Naoki

Watanabe, Kenichi

Watanabe, Shun

Watanabe, Yoshiko

## Y

Yamada, Tetsuya

Yamamoto, Takeshi

Yamamoto, Nobuko

Yamamoto, Naoto

Yasugi, Takumi

Yiu, Wai-Ki

Yokoyama, Naoyuki

Yokoyama, Kenichi

Yoshimura, Koichi

Yoshitake, Akihiro

Yoshizumi, Masao

